# Smoke-Free Homes and Home Exposure to Secondhand Smoke in Shanghai, China

**DOI:** 10.3390/ijerph111112015

**Published:** 2014-11-20

**Authors:** Pinpin Zheng, Carla J. Berg, Michelle C. Kegler, Wenjie Fu, Jing Wang, Xilan Zhou, Dong Liu, Hua Fu

**Affiliations:** 1 Key Laboratory of Public Health Safety, Ministry of Education, School of Public Health, Fudan University, Shanghai 200032, China; E-Mails: 11211020034@fudan.edu.cn (W.F.); hfu@fudan.edu.cn (H.F.); 2 Rollins School of Public Health, Emory University, Atlanta, GA 30322, USA; E-Mails: carlajberg@gmail.com (C.J.B.); mkegler@emory.edu (M.C.K.); 3 Center of Disease Control, Pudong District, Shanghai 200136, China; E-Mails: pdcdcjkjy@126.com (J.W.); zhouxlcarol@163.com (X.Z.); 4 Center of Disease Control, Fengxian District, Shanghai 201400, China; E-Mail: fxcdcliudong@126.com

**Keywords:** secondhand smoke, home, Shanghai, China

## Abstract

Few studies have examined home exposure to secondhand smoke (SHS) in China. This study aimed to document: (1) the prevalence and correlates of exposure to SHS in homes (in adult non-smokers) in Shanghai, and (2) enforcement of rules, harm reduction behaviors, and self-efficacy for maintaining smoke-free homes in Shanghai. A total of 500 participants were recruited using a multistage proportional random sampling design in an urban and suburban district to complete a survey. Among the total 355 nonsmokers, 127 (35.8%) participants reported being exposed to SHS in the past 7 days. Participants living with smokers in the home, with no smoking restriction at home, and having children younger than 18 were more likely to be exposed to SHS at home. Higher self-efficacy in maintaining a smoke-free home was negatively associated with home SHS exposure. Having visitors who smoke was the greatest policy enforcement challenge. Ineffective measures such as opening windows were more commonly used in homes with partial bans. Educational initiatives to protect against SHS exposure in the home should promote smoke-free homes, address challenges to implementing such policies, and address misconceptions regarding the effectiveness of supposed harm reduction behaviors.

## 1. Introduction

Secondhand tobacco smoke (SHS) is one of the most common and important sources of pollution in the indoor home environment. There is no safe level of exposure to SHS [1]. More than 4000 chemicals have been identified in SHS, with more than 50 of these known to cause cancer [1]. The link between SHS and several health outcomes, such as respiratory infections, ischemic heart disease, lung cancer, and asthma has long been established. It is estimated that more than 600,000 deaths per year worldwide are caused by SHS which is more than 1% of all deaths. Women and children are disproportionally affected by exposure to SHS [2].

Effective interventions for reducing SHS exposure include establishing smoke-free policies at work and in public places, as well as creating and maintaining smoke-free homes. Article 8 of the WHO Framework Convention on Tobacco Control (FCTC) urges policy makers to require by law that all indoor workplaces and public places be 100% smoke-free environments. In the past decades, in response to FCTC, an increasing number of countries have established smoke-free policies to eliminate SHS exposure in public places and work places. Households are considered private places and could not be protected under the FCTC. Therefore, even as smoke-free public policies are becoming more widespread, home exposure to SHS is still a prominent problem, particularly among vulnerable children and women [3].

More than 300 million smokers and 740 million nonsmokers suffer from SHS exposure in China, indicating its public health importance. It is estimated that 67.3% of nonsmokers are exposed to SHS in their homes [4], which is lower than the prevalence of exposure to SHS in public places (72.7%) but higher than that in work places (63%). A cross-country comparison of SHS exposure among adults in developing countries showed that exposure to SHS in the home was quite varied and ranged from 17.3% (Mexico) to 73.1% (Vietnam) [5]. One of the important reasons leading to high exposure to SHS in the home is that smoke-free home policies are not yet widely adopted. Prior research has documented the prevalence of smoke-free households in China varying from 6.3% to 26% [6,7,8].

Shanghai, the largest and the most developed metropolis in the mainland of China, has enforced the Shanghai Public Places Smoking Control Legislation since 2010. A former study has indicated that initial positive effects were achieved including decreased exposure to SHS among occupational employees [9]. However, little is known about the status of exposure to SHS in the home, particularly since the implementation of this legislation.

The objective of this study was to: (1) investigate the prevalence and correlates of exposure to SHS in homes (in adult non-smokers) in Shanghai, and (2) examine enforcement of rules, supposed harm reduction behaviors, and self-efficacy for maintaining smoke-free homes in Shanghai. It is expected that these findings would guide the development of home SHS exposure reduction intervention strategies in Shanghai and in China more broadly.

## 2. Methods

### 2.1. Setting

Two districts were purposively sampled in this study from a total of 17 districts in Shanghai. Pudong New Area, located in the east of Shanghai and regarded as China’s financial and commercial hub, was selected as the urban area. Fengxian District, located in the southern part of Shanghai and less economically developed, was selected as the suburban area in this study.

### 2.2. Sampling

Participants were recruited based on a multistage proportional random sampling design. In the first stage, 5 communities were randomly selected from each district. In the second stage, 250 households were randomly selected in these communities in each district through a proportional random sampling design. In the end, in each of the selected households, the person (aged ≥18 years) whose birthday was closest to the interview date was invited to participate in the study.

A total of 500 participants completed the questionnaire with a response rate of 87% from November 2012 to January 2013. Males accounted for 48.2% of the total sample. Participants aged 20–39 years accounted for 45.4%. Overall, 29% were current smokers (58.1% among men and 1.9% among women). There were 29.6% that reported monthly household income less than 3000 Yuan, 38.5% reported from 3001 to 5999 Yuan, 15.6% reported from 6000–7999 Yuan and 16.2% reported more than 8000 Yuan (as in Table 1).

Written consent was obtained from those participants who could read and write; others gave verbal consent. All data were collected using face-to-face interviews by trained students from Fudan University. The IRB of the Public Health School in Fudan University approved the protocol.

### 2.3. Measurement

A standardized questionnaire was used for data collection including information on demographics, composition of household, home exposure to SHS, current household smoking policy and its enforcement, actions to reduce SHS exposure, and self-efficacy for maintaining a policy.

Home exposure to SHS was measured among nonsmokers by asking: “During the past 7 days, on how many days have people smoked in your home in your presence?” Participants reporting one day and above were considered to be exposed to SHS at home.

Participants were asked, “Which statement best describes the rules about smoking inside your home? This does not include decks, garages, or porches” A complete smoke-free home was defined as “Smoking is not allowed anywhere inside your home” [10]. A partial policy was defined as “Smoking is allowed in some places or at some times.” For those participants with a complete smoke-free home policy, enforcement of such rules in six situations was assessed (listed in Table 2). Cronbach’s alpha in the current study for the scale was 0.87.

A range of potential SHS harm reduction behaviors were assessed by asking, “To reduce secondhand smoke in your home, please indicate how often you or the smoker in your home have done any of the following?” Some examples were “opened the window to let the smoke escape”, “only smoked in certain rooms”, and “smoked near a running fan.” Responses were provided on a scale of 1 = Never to 5 = Almost always. Cronbach’s alpha for this 10 item scale was 0.91.

To assess self-efficacy to maintain a smoke-free policy among all participants, participants were asked, “How certain are you/not certain that you could maintain a smoke-free home” in relation to five situations in which it might be difficult to maintain a smoke-free home such as “When guests who smoke visit?” (see Table 2). Participants responded on a scale of 1 = Not at all sure to 5 = Absolutely sure. Specifically among smokers, this group was asked about their self-efficacy to maintain a smoke-free home policy in seven situations such as “When I am alone with my child” and “When I am tired/it feels like too much effort” (listed in Table 2). Cronbach’s alpha for the all items and for smoker-specific items were 0.83 and 0.81, respectively.

Lifetime smokers were defined as those who had smoked at least 100 cigarettes in their lifetime, and current smokers were defined as those reporting smoking in the past 30 days. For smokers, the questionnaires also covered smoking-related information such as days of smoking in the past 30 days and quit attempts in the past 12 months.

### 2.4. Statistics

Fisher’s exact tests or χ^2^ tests were used to examine group differences for categorical variables (e.g., gender, education), and Student t-tests were used to examine differences between groups for continuous variables (e.g., days of smoking in the past 30 days). Binary logistic regression was used to investigate correlates of home exposure to SHS. Specifically, all variables that were associated with home SHS exposure in bivariate analyses at *p* < 0.10 were forced into the model including age group, work situation, whether have a smoker at home, number of friends/relatives who smoke, smoke-free policy level, and self-efficacy score. Exposure to SHS was considered among non-smokers. All data were analyzed by SPSS 21.0.

## 3. Results

Overall, 176 participants (35.3%) had complete smoke-free home policies, and 61.6% of participants had smokers in the home. Among the 355 nonsmokers, 127 (35.8%) reported being exposed to SHS in the home in the past 7 days. Among those with less than high school education, 31.9% had a complete policy in contrast to 45.6% with college degrees. Correspondingly, the prevalence of being exposed to SHS at home was 54.5% and 45.0% respectively. Similarly, Among those with less than 3000 Yuan monthly household income, 38.4% of them had complete smoke-free policy while among those with more than 8000 Yuan monthly household income, 47.5% had complete smoke-free policy.

Participants who had smokers at home had a higher prevalence of home exposure to SHS than those who did not live with smokers (65.9% *vs.* 21.4%, *p* < 0.001). Also, participants with children younger than 18 years old had a higher prevalence of exposure to SHS in the home than those who did not have children (51.4% *vs.* 46.5%, *p* = 0.003). Participants with complete smoke-free policies had lowest level of exposure to SHS (33.1%, *p* < 0.001), as presented in Table 1.

**Table 1:** Distribution of smoke-free home policy level and SHS exposure among participants.

Characteristics		Smoke-Free Policy Level				Exposed to SHS		
	Total (%) (N)	Complete (%)	Partial (%)	No (%)	*p*	Nonsmokers (%)	Yes (%)	*p*
*Sociodemographics*								
Gender								
Male	48.2 (241)	30.1	39.7	30.1	0.07	28.5	51.0	0.21
Female	51.8 (259)	40.1	33.9	26.1		71.5	46.7	
Ethnicity								
Han	99.6 (498)	35.2	36.8	27.9	0.55	99.4	48.8	0.67
Other ethnicities	0.4 (2)	50	0	50		0.6	50	
Age								
<29	25.6(128)	37.1	32.3	30.6		25.9	51.2	0.07
30–39	20.4 (102)	30.7	40.6	28.7		22.8	56.9	
40–49	19.6 (98)	36.1	38.1	25.8		19.4	48.0	
50–59	18.2 (91)	36.3	38.5	25.3		16.3	45.1	
≥60	16.2 (81)	36.2	35.0	28.8		15.2	38.3	
Education								
Less than high school	51.7 (259)	31.9	37.0	31.1	0.40	51.3	54.5	0.11
High school graduated	19.3 (97)	34.7	40	25.3		15.7	60.0	
Some college/technical college	12.8 (64)	38.1	36.5	25.4		13.7	50.8	
College graduate or higher	16.1 (80)	45.6	30.4	24.1		19.4	45.0	
Employment status								
Employed full-time	59.5 (298)	33.9	37.7	28.4	0.20	58.9	53.4	0.02
Employed part-time	9.9 (49)	29.2	37.5	33.3		7.7	53.1	
Retired	19.0 (95)	38.7	40.9	20.4		19.1	38.3	
Homemaker	7.5 (38)	35.1	18.9	45.9		10.3	32.4	
Monthly household income								
Less than 3000 Yuan	29.6 (148)	38.4	38.4	23.3	0.01	28.4	51.0	0.212
3001–5999 Yuan	38.5 (193)	28.4	40.0	31.6		36.6	52.9	
6000–7999 Yuan	15.6 (78)	31.2	29.9	39.0		16.5	46.8	
Monthly household income								
More than 8000 Yuan	16.2 (81)	47.5	33.8	18.8		18.5	38.8	
Marital status								
Married	86.6 (433)	35.7	37.6	26.6	0.34	87.9	48.8	0.285
Single	10.4 (52)	32.7	32.7	34.6		9.6	53.8	
Other	3.0 (15)	33.3	20	46.7		2.5	33.3	
Setting								
Urban	49.5 (248)	39.6	40.0	20.4	0.001	49.9	49.8	0.223
Suburban	50.5 (252)	30.8	33.6	35.6		50.1	48.0	
*Social factors*								
Smokers in the home								
Yes	61.6 (309)	25.2	44.4	30.4	<0.001	51.8	21.4	<0.001
No	38.3 (191)	51.6	24.2	24.2		48.2	65.9	
Have children under 18								
Yes	46.8 (234)	38.2	38.2	23.6	0.41	48.8	51.4	0.003
No	53.2 (266)	34.4	36.5	29.0		51.2	46.5	
Have children under 5								
Yes	25.5 (128)	44.7	30.1	25.2	0.04	26.8	49.3	0.745
No	74.5 (372)	32.3	39.0	28.7		73.2	48.0	
Number of friends that smoke								
Less than half	52.4 (262)	42.5	35.1	22.4	0.001	40.8	42.1	0.001
Half or more	47.6 (238)	27.2	38.7	34.0		59.2	56.5	
*Smoking status*								
Currrent smokers	29.0 (145)	21.0	46.2	32.9	0.001		/	/
Non-smokers	71.0(355)	41.1	32.9	26.1			35.8	
*Smoke-free policy factors*								
Level of smoke-free home policy		/	/	/				
Complete smoke-free policy	35.3 (176)						33.1	<0.0001
Partial smoke-free policy	36.7 (184)						54.4	
No smoke-free policy	28.0 (140)						59.7	
Total self-efficacy score ^a^	15.69 ± 6.09	18.31 ± 5.92	14.80 ± 5.14	13.72 ± 6.31	0.001	16.58 ± 6.07	13.66 ± 5.61	<0.001
* Among smokers	M ± SD	M ± SD	M ± SD	M ± SD	*p*			
Days of smoking, past 30 days	26.1 ± 7.91	23.88 ± 9.45	25.61 ± 8.18	27.89 ± 6.41	0.09	/	/	/
Quit attempts, past 12 months	1.92 ± 4.01	3.13 ± 7.48	1.44 ± 1.93	1.81 ± 2.80	0.17	/	/	/

^a^ Score for all participants reported in M ± SD; see Table 2 for items. * These questions were only asked among the smokers.

The greatest enforcement challenge to implementing a complete policy was “Visitors break the rule by smoking” (57.1%), followed by “Someone in the household finds it difficult to ask smokers to obey the rule” (41.1%; see Table 2). People with partial smoke-free policies had a higher score for harm reduction behaviors than others (*p* < 0.01). Among the participants with complete smoke-free home policy, the most common step to reduce SHS exposure was “Talked about making home smoke-free” while among those with partial or no rules, the most important action was “Opened window to let smoke escape.” Participants who already enforced a complete smoke-free home policy had higher self-efficacy scores than those with partial or no bans. In addition, participants exposed to SHS at home had lower self-efficacy scores in maintaining a smoke-free home than those who were not exposed to SHS at home (17.41 ± 5.93 *vs.* 13.33 ± 5.61, *p* < 0.001). The most prominent challenge to self-efficacy to maintain a smoke-free policy among all participants was when older relatives or guests who were smokers visit. For smokers, a possible cue to break the smoking rules was drinking alcohol.

Table 3 shows the final logistic regression model indicating significant correlates of home exposure to SHS. Having smokers at home, no smoking restriction or kids less than 18 were positively associated with SHS exposure. People with lower self-efficacy scores to maintain a smoke-free home were more likely to be exposed to SHS in the home.

**Table 2:** Enforcement, supposed harm reduction behaviors, and self-efficacy of maintaining policy related to smoking in the home in relation to level of smoke-free home policy, and exposure to SHS in the home in the past 7 days.

Questions	Smoke-Free Home Policy Level			Exposed to SHS	
	Complete	Partial	No	No	Yes
Enforcement of complete policy ^a^	%	%	%	%	%
Someone in the household breaks the rules by smoking	34.3	/	/	26.4	45.7 *
Someone in the household finds it difficult to ask smokers to obey the rule	41.1	/	/	30.3	57.1 **
Visitors break the rule by smoking	57.1	/	/	49.1	60.0
The rule is broken when those who set it up are not around	32.8	/	/	26.4	37.1
It’s difficult to find a place to smoke outside the household	30.3	/	/	24.5	37.1
Someone who quit started smoking again	35.4	/	/	28.2	42.9
Supposed harm reduction behaviors ^b^	M ± SD	M ± SD	M ± SD	M ± SD	M ± SD
Opened window to let smoke escape	3.71 ± 1.26	3.91 ± 0.93	3.79 ± 1.96	3.82 ± 1.19	3.76 ± 0.96
Only smoked in certain rooms	2.98 ± 1.53	3.57 ± 1.20	2.96 ± 1.37 **	3.32 ± 1.47	2.94 ± 1.37 *
Smoked near a running fan	2.50 ± 1.54	2.88 ± 1.29	2.40 ± 1.24 **	2.51 ± 1.51	2.64 ± 1.23
Reduced number of cigarettes smoked inside the home	3.42 ± 1.43	3.53 ± 1.07	2.97 ± 1.72 **	3.39 ± 1.35	3.36 ± 1.12
Only smoked indoors when no one is home	2.89 ± 1.49	3.01 ± 1.11	2.69 ± 1.25	2.66 ± 1.40	2.86 ± 1.16
Only smoked indoors when the children were gone	2.72 ± 1.47	3.11 ± 1.21	2.71 ± 1.29 *	2.61 ± 1.40	2.95 ± 1.23 *
Used nicotine replacement therapy like nicotine gum or patch	2.66 ± 1.46	2.22 ± 1.19	2.03 ± 1.28 **	2.29 ± 1.37	2.22 ± 1.24
Left the room to have a cigarette	3.26 ± 1.38	3.16 ± 1.22	2.83 ± 1.20 *	3.16 ± 1.35	3.12 ± 1.22
Used an air freshener to get rid of the smoke or smell	2.40 ± 1.43	2.24 ± 1.20	2.10 ± 1.32	2.34 ± 1.37	2.06 ± 1.15
Talked about making home smoke-free	4.48 ± 2.73	2.97 ± 1.98	3.14 ± 2.60 **	4.00 ± 2.69	3.53 ± 2.55
Total score for harm reduction behaviors	29.00 ± 10.62	30.09 ± 6.70	26.31 ± 9.14 **	28.26 ± 9.81	28.80 ± 7.93
Self-efficacy for maintaining policy ^c, d^	M ± SD	M ± SD	M ± SD	M ± SD	M ± SD
**All participants**					
When it is raining outside	3.87 ± 1.39	3.05 ± 1.22	2.82 ± 1.41 **	3.64 ± 1.36	2.76 ± 1.33 **
When it is cold outside	3.86 ± 1.38	3.07 ± 1.19	2.88 ± 1.41 **	3.66 ± 1.33	2.76 ± 1.31 **
When guests who smoke visit	3.29 ± 1.46	2.75 ± 1.25	2.94 ± 1.38 **	3.18 ± 1.41	2.43 ± 1.25 **
When older relatives or someone you respect who are smokers visit	3.24 ± 1.43	2.72 ± 1.28	2.53 ± 1.36 **	3.14 ± 1.38	2.44 ± 1.29 **
When it is dark outside	3.89 ± 1.32	3.23 ± 1.17	2.94 ± 1.40 **	3.73 ± 1.27	2.88 ± 1.29 **
**Smokers only**					
When I am alone with my child	4.20 ± 1.16	3.39 ± 1.27	2.89 ± 1.40 **	3.50 ± 1.34	3.30 ± 1.38
When I am feeling lazy	3.38 ± 1.27	2.86 ± 1.09	2.83 ± 1.29	3.05 ± 1.20	2.86 ± 1.20
When I am alone	3.40 ± 1.38	2.84 ± 1.10	2.51 ± 1.35 *	2.89 ± 1.28	2.80 ± 1.28
When no one cares if I smoke indoors	3.50 ± 1.13	2.82 ± 1.04	2.68 ± 1.37 **	2.97 ± 1.21	2.86 ± 1.20
When I am drinking alcohol	3.10 ± 1.37	2.73 ± 1.12	2.81 ± 1.38	2.92 ± 1.24	2.73 ± 1.29
When I am in a hurry	3.40 ± 1.33	2.91 ± 1.13	2.91 ± 1.43	3.08 ± 1.31	2.92 ± 1.27
When having coffee or tea	3.63 ± 1.27	3.00 ± 1.23	2.83 ± 1.39 *	3.12 ± 1.36	3.00 ± 1.29
Total score for all participants	18.31 ± 5.92	14.80 ± 5.14	13.72 ± 6.31 **	17.41 ± 5.93	13.33 ± 5.61 **
Total score for smokers	24.72 ± 7.45	20.66 ± 5.58	19.31 ± 7.59 **	20.44 ± 6.55	21.63 ± 7.35
Days of exposure among nonsmokers	0.82 ± 1.85	1.75 ± 2.25	2.24 ± 2.91 **	0 **	3.87 ± 2.54 **

* p < 0.05; ** *p* < 0.01; ^a^ Response options of no or yes; ^b^ On a scale of 1 = Never to 5 = Almost always; ^c^ On a scale of 1 = Not at all sure to 5 = Absolutely sure; ^d^ Total score for Self-efficacy for maintaining policy is the sum scores of items designed for all participants.

**Table 3:** Multiple logistic regression model indicating correlates of exposure to SHS in the home in the last 7 days.

Predictors	Adjusted OR	95% CI	*p*
Age group			0.32
<39	Ref	--	--
40–59	0.96	0.50–1.96	0.99
≥60	1.07	0.27–4.35	0.92
Work situation			0.35
Employed full-time	Ref	--	
Employed part-time	0.74	0.24–2.27	0.61
Retired	0.41	0.12–1.38	0.15
Homemaker	0.25	0.03–1.11	0.23
Have a smoker in home	8.50	4.40–16.40	0.001
With kids less than 18	2.06	1.10–3.85	0.023
Number of friends/relatives who smoke			0.37
Less than half	Ref	--	
Half or more	1.33	0.72–2.42	0.37
Smoke-free home policy level			0.05
Complete policy	Ref	--	
Partial policy	1.31	0.64–2.69	0.46
No policy	2.63	1.18–5.86	0.018
Self-efficacy score (all participants)	0.89	0.85–0.94	<0.001

Note: All variables that were associated with policy status in bivariate analyses at *p* < 0.10 were forced into the model according to the Table 1 bivariate analyses results regarding SHS exposure.

## 4. Discussion

Increased awareness among the public about the harms of SHS has led to policies prohibiting smoking in public places and work places. There is some evidence that those subject to bans in workplaces are more likely to have bans in their homes [11,12]. Private homes, the enclosed environment where people generally spend the most time, and only voluntary rules are possible, have become a primary source of exposure to SHS among children and nonsmoking adults [13]. There is evidence from a small number of countries that individuals, including many smokers, are increasingly making their homes smoke-free [14,15].

The current study documented a prevalence of home exposure to SHS of 35.8%, lower than the average national level which may reflect changes coinciding with the provincial smoke-free legislation. According to a social diffusion perspective, smoke-free public places seem to stimulate adoption of smoke-free homes [16,17]. Our previous research also confirmed increased awareness of the harm of SHS and support of smoke-free policies after the enforcement of legislation in Shanghai, which may become triggers of setting up smoke-free home policies [9]. Further efforts should be engaged to encourage the establishment of smoke-free home policies to protect the health of nonsmokers and children, particularly when public smoke-free policies are implemented to help thrust the momentum of change forward.

This study confirmed that having a smoke-free home policy was negatively associated with exposure to SHS in the home, which is consistent with other studies [18,19,20]. Restrictions were more common when there were no smokers in the home and when there were children present [14,21] especially among children less than 5 years old [10]. It is surprising that having children less than 18 was positively associated with exposure to SHS in the home, which indicates that adolescents may be seriously exposed to SHS. Other research has shown that having children less than 5 years of age was a correlate of having a smoke-free home policy while having children under 18 years old was not [10]. In addition, studies have shown that pregnancy and giving birth may serve as impetuses to establishing a smoke-free home [22,23]. Another important consideration is that exposure to SHS could increase the likelihood of poor academic performance and a greater number of school days missed due to illness as well as the possibility of adolescents smoking [24,25]. Education regarding the impact of SHS exposure on children and adolescents may impact future family practices around smoking in the home. Although not significant, our result also indicated that participants which higher socioeconomic level seemed more likely to have smoke free policy and suffer less SHS exposure at home, which is consistent with results from other studies [26,27]. The findings highlight the need to address disparities by socioeconomic status when promoting smoke-free homes.

Even among participants with a complete smoke-free home policy, 33.1% were exposed to SHS at home in the past week which implies enforcement challenges of such policies. Older relatives and other smokers visiting were the most important barriers to enforcing and maintaining such a policy. The demands of visitors are given priority in Chinese culture, and some people provide guests with cigarettes. One study in six counties in China showed that 42.1% of nonsmokers would offer cigarettes to guests [7]. This may make a smoke-free home policy more difficult to be implemented and/or enforced. According to our findings, this situation is more difficult when there is a smoker in the home. Also, finding it difficult to ask smokers to obey a smoke-free policy is another obstacle in enforcement. Health interventions to reduce SHS should address the Chinese value system in which elder generations and visitors are highly respected. Informing guests about the household rules through explicit notification such as smoke-free signs may be feasible measures to address the policy and avoid embarrassment among visiting smokers.

Living with smokers is a typical barrier to establishing smoke-free home. However, smoke-free homes may also help smokers gain greater control over nicotine dependence. Data has shown that smoke-free homes have been associated with increased cessation among smokers, decreased cigarette consumption [28], and reduced likelihood of adolescent smoking [25].

Actions to reduce SHS or supposed harm reduction behaviors were common in this sample, especially among those with partial bans. In particular, opening a window to let smoke escape and only smoking in certain rooms were common among participants with partial bans. For the participants with a complete policy, the most important action was “talked about making home smoke-free”. Participants with no rules had the lowest score for these actions. Compared to those without smoking restrictions in the home, participants with partial ban were more likely to adopt some supposed harm reduction behaviors, such as smoking near a running fan or smoking indoors when children were not present. Further education should focus on the limitations of these actions and encourage more households to be smoke-free.

Self-efficacy for maintaining a smoke-free home was negatively associated with exposure to SHS at home. Participants who already had a smoke-free home policy had the highest scores while those having no restrictions had the lowest. Informing the public about the benefits of a smoke-free home and providing possible solutions to the barriers could improve self-efficacy in maintaining a smoke-free home, which theoretically would promote the implementation of a smoke-free home.

Several limitations in this study need to be addressed. This survey relied on self-report by participants, which may lead to some recall and social desirability bias. A limitation of this study is a reliance on self-reported measures of exposure to SHS in homes. Objective measures, such as air nicotine monitors should be considered in future studies. As a cross-sectional study, there is uncertainty regarding the temporal sequence between the factors of interest, such as SHS exposure, establishing a smoke-free home, and social factors related to rules about smoking in the home. Furthermore, there may be some significant difference in SHS exposure and home smoking rules between those who agreed to participate in this study and those who declined to participate. Despite the limitations, this study provides empirical evidence for the need to address SHS exposure in the home in Shanghai and in China more generally.

## 5. Conclusions

Due to the fact that homes are a substantial source of SHS for children and nonsmoking adults, strategies to successfully reduce exposure to home SHS are needed. Because households cannot be required to go smoke-free by legislation, clinical and educational initiatives are the only viable direct approaches for reducing SHS exposure in this setting [29]. Active and determined actions should be promoted through advice from physicians and mass media campaigns to implement and maintain smoke-free households. Special considerations regarding misconceptions about harm reduction behaviors and the particular barriers to enforcement within the Chinese culture should be made. Finally, using the implementation of public smoke-free policies as an impetus for promoting smoke-free homes may prove to be effective.
